# Kinematic Analysis of Human Gait in Healthy Young Adults Using IMU Sensors: Exploring Relevant Machine Learning Features for Clinical Applications

**DOI:** 10.3390/bioengineering11020105

**Published:** 2024-01-23

**Authors:** Xavier Marimon, Itziar Mengual, Carlos López-de-Celis, Alejandro Portela, Jacobo Rodríguez-Sanz, Iria Andrea Herráez, Albert Pérez-Bellmunt

**Affiliations:** 1 Bioengineering Institute of Technology, Universitat Internacional de Catalunya (UIC), 08195 Barcelona, Spain; xmarimon@uic.es (X.M.); aeportela@uic.es (A.P.); i.herraez@uic.es (I.A.H.); 2 Automatic Control Department, Universitat Politècnica de Catalunya (UPC-BarcelonaTECH), 08034 Barcelona, Spain; 3 Institut de Recerca Sant Joan de Déu (IRSJD), 08950 Barcelona, Spain; 4 ACTIUM Research Group, Universitat Internacional de Catalunya (UIC), 08195 Barcelona, Spain; carlesldc@uic.es (C.L.-d.-C.); aperez@uic.cat (A.P.-B.); 5 Institut Universitari d’Investigació en Atenció Primària (IDIAP Jordi Gol), 08007 Barcelona, Spain

**Keywords:** gait, walking, gait analysis, artificial intelligence, machine learning, falls, orthesis

## Abstract

Background: Gait is the manner or style of walking, involving motor control and coordination to adapt to the surrounding environment. Knowing the kinesthetic markers of normal gait is essential for the diagnosis of certain pathologies or the generation of intelligent ortho-prostheses for the treatment or prevention of gait disorders. The aim of the present study was to identify the key features of normal human gait using inertial unit (IMU) recordings in a walking test. Methods: Gait analysis was conducted on 32 healthy participants (age range 19–29 years) at speeds of 2 km/h and 4 km/h using a treadmill. Dynamic data were obtained using a microcontroller (Arduino Nano 33 BLE Sense Rev2) with IMU sensors (BMI270). The collected data were processed and analyzed using a custom script (MATLAB 2022b), including the labeling of the four relevant gait phases and events (Stance, Toe-Off, Swing, and Heel Strike), computation of statistical features (64 features), and application of machine learning techniques for classification (8 classifiers). Results: Spider plot analysis revealed significant differences in the four events created by the most relevant statistical features. Among the different classifiers tested, the Support Vector Machine (SVM) model using a Cubic kernel achieved an accuracy rate of 92.4% when differentiating between gait events using the computed statistical features. Conclusions: This study identifies the optimal features of acceleration and gyroscope data during normal gait. The findings suggest potential applications for injury prevention and performance optimization in individuals engaged in activities involving normal gait. The creation of spider plots is proposed to obtain a personalised fingerprint of each patient’s gait fingerprint that could be used as a diagnostic tool. A deviation from a normal gait pattern can be used to identify human gait disorders. Moving forward, this information has potential for use in clinical applications in the diagnosis of gait-related disorders and developing novel orthoses and prosthetics to prevent falls and ankle sprains.

## 1. Introduction

Walking is the most natural mode of locomotion in humans to travel independently and in an efficient manner and it has proven health benefits for older adults. Walking has essential benefits for physical and psychological health [1,2]. The WHO points out that walking 30 min a day is enough for our body to obtain the improvements associated with this practice. According to Perry and Burfield, the human gait cycle consists of two primary phases, the stance and swing phase, each consisting of several functional periods. The stance phase encompasses the following functional periods: loading response, midstance, terminal, and preswing. On the other hand, the swing phase is divided into initial swing, mid swing, and terminal swing [3]. In general, human gait can be defined as movement consistent with translation for the whole body permitted by a repetition of movements of body segments while maintaining balance. This procedure is accompanied by movement processes automatically controlled by the brainstem and spinal cord [4].

Although the study of gait has been of interest since ancient times [5], the development of gait analysis techniques (also known as walking or motion analysis) has experienced its greatest development in the last century. Studies involving high-quality motion analysis systems have provided substantial insight into human gait patterns, not only of healthy subjects but also those with various movement disorders [6,7]. This has led to numerous investigations related to how gait disturbance can be a predictor of specific pathologies [8].

Injuries resulting from falls in older adults represent a significant health burden [9]. The frequency of falls increases with age and the fragility level. Between 32–42% of people aged 70 years and over fall each year [10,11,12]. About one-third of these individuals are estimated to experience one or more falls each year, while 10% experience multiple falls annually [12]. Fall-related injuries are a healthcare problem associated with subsequent disability, hospitalization, morbidity, and mortality [13]. Approximately 1% of healthcare costs are fall-related expenditures in high-income countries [14]. For young individuals, lateral ankle sprains are another crucial condition related to gait problems. The peak incidence of ankle sprains occurred between the ages of fifteen and nineteen (7.2 per 1000 person-years) [15]. It is estimated that upward 45% of all sports-related injuries involve an ankle sprain [16,17,18]. Ankle injury rates range from 0.82 to 1.65 injuries per 1000 athletes-exposures depending on the sport [19].

The study of normal gait remains fundamental and relevant, whether it is driven by the interest in describing gait using new technological tools for clarifying the neuromuscular and biomechanical manifestations of the pathology, or for the purpose of designing intelligent orthoses that prevent the consequences of falls or the effects of ankle sprains. Many studies related to human gait analysis use Inertial Measurement Units (IMUs) sensors equipped with gyroscopes and accelerometers to measure spatio-temporal [20], kinematics [21], and kinetics features [22]. These small-size, affordable, energy-efficient, and easy-to-use sensors may play an important role in biomechanics fields due their fast processing and measurements during the physical activity [23,24]. The present study aimed to identify the key features of normal human gait using inertial unit (IMU) recordings in a walking test. Although there are many studies where inertial units are used to analyse normal human gait, each employs different features or markers to assess gait performance [25,26,27,28,29,30,31,32,33,34,35,36]. The current literature lacks concise studies that specify which key features may be used to optimally distinguish the most relevant events in human gait. Noteworthy examples include studies that utilize different kinds of features, such as the filtered acceleration and gyroscope data itself without feature extraction [23], spatiotemporal parameters [20,26], or biological outcomes (e.g., accelerations, stability, regularity, etc.) [33]. This study employed advanced statistical methods to determine the most relevant statistical features to consider in analysing acceleration and gyroscope signals from IMU devices. Understanding the key characteristics of a normal gait pattern is crucial, as any gait disorder will modify this normal pattern.

## 2. Materials and Methods

### 2.1. Study Design

A cross-sectional study was conducted at the Functional Anatomy Lab at the Universitat Internacional de Catalunya. The study procedures were conducted following the Declaration of Helsinki (World Medical Association, 2013).

### 2.2. Participants

A total of 32 participants (age range 19–29 years) were recruited for this study. In the context of our gait-related research conducted on the university campus, participants were recruited through university-wide communication channels, such as email newsletters, bulletin boards, and social media platforms, to invite students, staff, and faculty members. The inclusion criterion for selecting volunteers stipulated that they did not exhibit issues pertaining to movement or ambulation. Exclusion criteria included inability or difficulty walking at the indicated speeds, surgical intervention in the lower limb within the past five years, history of muscle or ligament injury in the lower extremity, and any musculoskeletal or neurological injury that alters or could alter ambulation.

### 2.3. Data Collection

**Figure 1:**
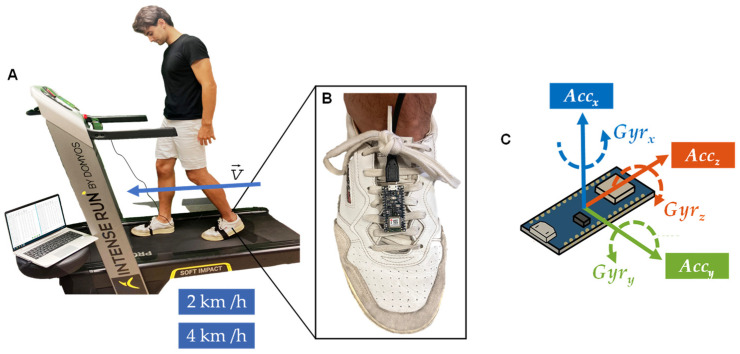
(**A**). A participant performing the experimental protocol. (**B**). Position of the Arduino in the forefoot area. (**C**). Arduino’s IMU sensor axis orientation.

Descriptive data and anthropometric measurements were collected from each participant, including age, gender, body weight, body mass, and leg dominance. These measurements are presented in Table 1. In our study, we defined leg dominance as the preferred or dominant leg that an individual naturally employs for activities such as kicking a ball or taking a step forward. The numerical data, including age, body weight, and body height, exhibited normal distributions in both subsets, categorized by gender and leg dominance. No significant differences in age were observed in either subset. However, gender disparities were observed in body mass and body height, whereas leg dominance did not show any notable differences. Dynamic data were collected using an Arduino Nano 33 BLE Sense Rev2 microcontroller board (Arduino, Monza, Italy) [25,26] positioned in the forefoot area of each foot [27,28,29,30] (refer to Figure 1B). The microcontroller board was equipped with IMU sensors (BMI270) capable of detecting acceleration with a three-axis accelerometer (±2 g acceleration scale and 0.06 mg/LSB of resolution) and gyroscope data [31,32,33]. Integrating the IMU sensor onto the same microcontroller board benefits us by mitigating electromagnetic interference, a challenge that tends to be more pronounced when dealing with standalone accelerometers connected via external wiring. The default factory calibration settings were found to be suboptimal. Therefore, we conducted a calibration of the IMU system using the protocol outlined by Tedaldi [37] to ensure the accuracy and reliability of the acquired data. Our experience has underscored the importance of regular recalibrations, conducted either weekly or after every five experiments, whichever occurs first, to mitigate performance deterioration resulting from component drift and aging.

Acceleration sensors detected changes in the Arduino’s velocity in their three main axes, whereas the gyroscope sensors detected the changes in the angular velocity. The orientations of the three axes are shown in Figure 1C. Acceleration in *x*-axis (AccX) detects velocity changes in the sagittal plane, with forward movements registered as positive and backward movements as negative. Gyroscope in the *x*-axis (GyrX) detects as positive the clockwise movement. Acceleration in the *y*-axis (AccY) indicates velocity variations in the frontal plane, cataloging movements to the right as positive and movements to the left as negative. Gyroscope in the *y*-axis (GyrY) detected as positive the counterclockwise movement. Finally, the acceleration in the *z*-axis (AccZ) registered upward movements as positive and as downward negative. Gyroscope in the *z*-axis (GyrZ) detected as positive the counterclockwise movement. Planes were described in an inclined plane as is the forefoot. Clock or counterclockwise is based on their respective acceleration vector. Considering the placement of the Arduino on the subject instep, this factor influences the data axes.

### 2.4. Procedure

The experimental protocol comprised two distinct parts: a 30-s forward walk on a treadmill (Domyos Intense Run, Decathlon, Villeneuve-d’Ascq, France) at a speed of 2 km/h, followed by an uninterrupted 30 s at 4 km/h. (refer to Figure 1A).

Throughout the entire experiment, participants were given specific instructions to wear suitable sports clothing and shoes. The Arduino board was secured on the forefoot of each participant. We established a USB connection between each Arduino board and the Coolterm software (Coolterm 2.0, The Meiers) (refer to Figure 1A) to ensure accurate data collection.

To prevent any interference with the natural gait of the participants during the forward walk, we employed 3-m cables. After data collection, we exported the acquired data for further processing and analysis using a custom script in MATLAB 2022b (Mathworks, Boston, MA, USA).

### 2.5. Data Processing

Acceleration and gyroscope data from the left and right legs were collected and processed using a custom script (MATLAB 2022b, Mathworks, Boston, MA, USA). The data were sampled at a frequency of F_s_ = 73.5 Hz. To reduce low-frequency movements during gait, a 5th order, low-pass Butterworth filter with a cutoff frequency of 4 Hz was used.

The magnitude of the filtered acceleration and gyroscope data was computed for each leg. The magnitude represents the magnitude of the acceleration or angular velocity vector. By comparing the magnitudes of the left and right leg data, the delay between the signals was estimated, allowing for their alignment.

**Figure 2:**
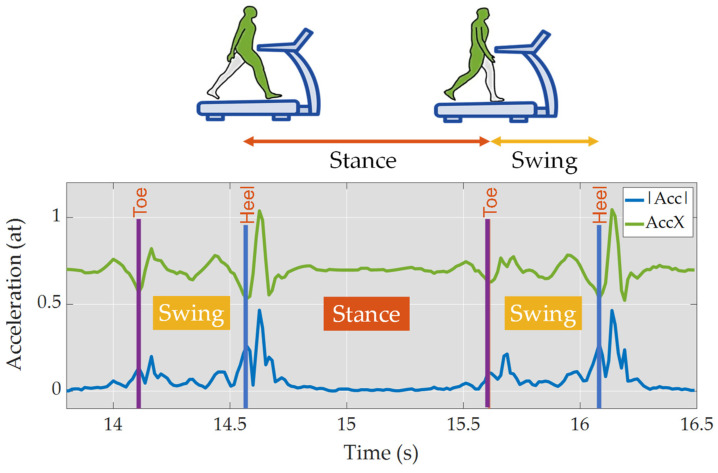
Gait representation based on acceleration magnitude (blue) and *x*-axis acceleration (green) at 2 km/h.

Next, we selected 25 s out of the total 30 s of data for each speed (2 km/h and 4 km/h), removing 2.5 s from each lateral (start and end), to be used in the study. In order to label specific gait events, the Signal Labeler tool provided by MATLAB was utilized (see an Example in Figure 2).

The Heel Strike label indicates the moment when the heel of the foot first makes contact with the ground during a gait cycle, while the Toe-Off label represents the moment when the toes leave the ground. Additionally, to precisely identify the locations of these events, we incorporated an FSR (Force-Sensing Resistor) sensor (OpenSmart FSR Sensor, Shenzhen, China) positioned at Area 2 of the foot chart to determine Toe-Off and Area 40 of the foot chart to specify Heel Strike [34]. The peaks detected from the FSR sensor provided us with precise information regarding the occurrence of these gait events.

Based on the labeled Heel Strike and Toe-Off events, the Swing Phase and Stance Phase were determined. The Swing Phase was defined as the period from Toe-Off to Heel Strike, during which the foot swings forward in preparation for the next step. Conversely, the Stance Phase was defined as the period from Heel Strike to Toe-Off, during which the foot remains in contact with the ground.

In order to perform feature extraction, various statistical features were computed on the data: gyroscope, acceleration in their three axes, and their magnitude. The magnitude of the acceleration was described as follows:

(1) $$\left|Acc\right|=\sqrt{Acc_{x}^{2}+Acc_{y}^{2}+Acc_{z}^{2}}$$

And the magnitude of the gyroscope was described in a similar way, as:

(2) $$\left|Gyr\right|=\sqrt{Gyr_{x}^{2}+Gyr_{y}^{2}+Gyr_{z}^{2}}$$

These features included mean, standard deviation, median, interquartile range, minimum, maximum, skewness, and kurtosis [35,36]. To segment the data, a time window approach was utilized. Each time window consisted of seven consecutive samples, and subsequent windows were incremented by four samples. This step allowed for the extraction of relevant features within distinct movement phases.

The selection of the time window size was driven by the need to obtain a sufficient number of samples to accurately identify stance and swing phases. Specifically, it was required to meet the minimum sample counts for confident phase identification at different walking speeds. We labeled both stance and swing phases, as illustrated in Figure 2, allowing us to determine the necessary number of samples for each phase. For example, during the stance phase at 4 km/h, a minimum of 38 samples (corresponding to the lower confidence interval) was necessary to achieve a 99.7% confidence level for the mean value. At a speed of 2 km/h, the minimum sample count for the swing phase remained consistent at 4 km/h, totaling 21 samples.

The stance phase was divided into four subphases: loading response, midstance, terminal, and preswing. For accurate characterization of each subphase in the future, it was necessary to have 9–10 samples per subphase. Similarly, the swing phase was divided into three subphases: initial swing, midswing, and terminal swing, and requires seven samples per subphase.

To ensure sufficient coverage of each subphase and avoid excessive repetition in the time window selection, the time window of seven samples with an increment of four samples was chosen as it provided a reasonable balance between capturing the necessary data and minimizing redundancy.

### 2.6. Feature Selection

The selection of features was carried out based on two main considerations: statistical tests and feature correlation. Two different thresholds were explored, employing the ANOVA test and the Post-Hoc test, which were computed using Jamovi version 2.3.21 (The jamovi project, 2023, Sydney, Australia), a GUI interface based on the *R* language. Initially, normality tests were conducted on the features. Visual inspection indicates sufficiently normal distributions considering the robustness of ANOVA to non-normal error distributions [38].

The first option for feature selection was the one-way Welch’s ANOVA test since we compared the categorical feature Phase (and/or Event), which has more than two subgroups (Heel Strike, Stance, Toe-Off, and Swing), with each quantitative feature measured using IMU sensors. The Welch Method was used because of unequal variances between subgroups in a quantitative feature, i.e., standard deviation in Stance and Swing phases. Alternatively, the Tukey HSD Post-Hoc Test was employed to analyze the differences between each pair of phases, in case the F-test was significant. The Tukey test is used to compare two means as it protects against the inflated risk of type I error arises when several significance tests are performed after ANOVA.

For this initial analytical approach, we had two sets of features: ANOVA and Post-Hoc features. The ANOVA features were selected from those with a *p*-value from the F-test lower than 0.01 across all subgroups (Heel Strike, Stance, Toe-Off, Swing). On the other hand, the Post-Hoc features were identified through the Tukey HSD Post-Hoc Test. These features demonstrated a significant pairwise difference, indicated by a *p*-value lower than 0.05, in the comparisons of Stance-Swing and Toe-Off-Heel Strike. Moreover, to ensure specificity, these features also exhibited *p*-values higher than 0.05 in all other pairwise comparisons.

Subsequently, the second approach focused on establishing a threshold for feature correlation with the intention of selecting the quantitative features that exhibited less correlation than the specified threshold. Pearson Correlation coefficients were employed for the correlation calculations. The feature selection filter operated using both the correlation matrix and the ranking of features based on F-test importance. This process encompassed all quantitative features. For instance, at a threshold of 80%, if two features exhibited a correlation of 0.83, the filter retained the feature with the highest importance according to the F-test and discarded the other. This process iterated across all features until none of them surpassed the 0.80 correlation threshold.

We had a total of 22 possibilities to consider, meaning 22 sets of features, comprising two tests per 11 correlation thresholds. To determine the most suitable approach, we considered the number of features used and the resulting accuracy obtained using a Narrow Neural Network. By evaluating these factors, we aimed to identify the optimal approach for feature selection, as well as to identify the most relevant features for our study. More comprehensive information will be provided in Section 3.1 of the Results.

### 2.7. Spider Plots

To assess the quantitative variables of the most relevant features in each phase, spider plots were utilized. Separate spider plots were generated for the data captured at 2 km/h and 4 km/h, as well as a combined spider plot including both datasets, referred to as the Walk data. Each spider plot represented the characteristics of features during both phases (Swing and Stance) and events (Heel Strike and Toe-Off).

From each spider plot, four distinct areas were calculated and obtained: Heel area, Stance area, Toe area, and Swing area. These areas provided insights into the distribution and magnitude of the variables within each phase and event. Additionally, a bar plot was created to display the ratios between velocities, along with the corresponding areas. This visualization allowed for a comparative analysis of the areas and their relationship to the different walking velocities. To explore the clinical applications and delve into the interpretation of the results, please refer to Section 4.

### 2.8. Machine Learning Analysis

This section aims to identify manually labelled gait events (Toe-Off and Heel Strike) and phases (Stance and Swing) by using different artificial intelligence classifier models fed with the statistical features extracted automatically from the gyroscope and acceleration signals. Several classifier models, including decision trees, support vector machines (SVM), Neural Networks (NNs), and *k*-nearest neighbor algorithms (KNN), were computed and evaluated. Many studies rely on machine learning methods for the recognition process. Decision trees (DT) and k-nearest neighbor (KNN) algorithms are commonly used to identify gait phases and disorders [23,39,40]. For analyzing and sorting different activities such as running, jumping, or walking, support vector machines (SVM) are usually employed [23,41].

The machine learning analysis was performed using two different sets of features from the labelled gait events: the features selected through the feature selection method and the complete set of 64 features initially considered. A 5-fold cross-validation was implemented for each machine learning model to mitigate the risk of overfitting. The performance of the models was assessed by quantifying their validation accuracy, which represents the percentage of correctly detected gait events. By comparing, in terms of value, the performance of the models using these two feature sets, we aimed to evaluate the impact of feature selection on the predictive power of the models.

## 3. Results

### 3.1. Feature Reduction

**Figure 3:**
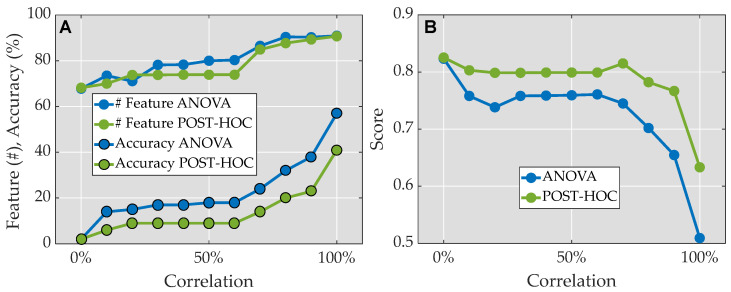
The selection of features is based on correlation thresholds (*x*-axis) derived from the ANOVA test (blue) and the POST-HOC test (green). (**A**). Plot of the accuracies achieved using a Narrow Neural Network, along with the corresponding number of features used. (**B**). Plot showing the ratio between the normalized accuracy achieved using a Narrow Neural Network and the absolute value of the normalized number of features, with a subtraction of −1 to obtain values closer to 1 for fewer features and closer to 0 for more features.

The accuracy of the Neural Networks consistently increased in both sets (based on ANOVA test threshold and Post-Hoc test threshold) as the correlation threshold was progressively raised (refer to Figure 3A). As we increased the correlation threshold, more features were included, leading to improved accuracy in the predictions. Among the correlation thresholds tested, the Post-Hoc test at 0% correlation demonstrated the highest ratio of accuracy to features, with a value of 0.83 (see Figure 3B). Analyzing the Post-Hoc test results, an increase in correlation (and thus feature inclusion) led to a decrease in the ratio, except for a notable peak at 70% correlation, where the ratio reached 0.82. Additionally, when examining the ANOVA test, lower correlations resulted in a reduced ratio, which then stabilized at 0.76 from 30% to 60% correlation. However, beyond 60% correlation, the ratio declined steadily, reaching almost 0.5 at 100% correlation.

After analyzing the results, the Post-Hoc test at 70% correlation was chosen as the preferred approach. This particular test yielded a total of 14 features for accurately predicting gait events at both 2 km/h and 4 km/h. The achieved accuracy using these features was 84.7%.

**Figure 4:**
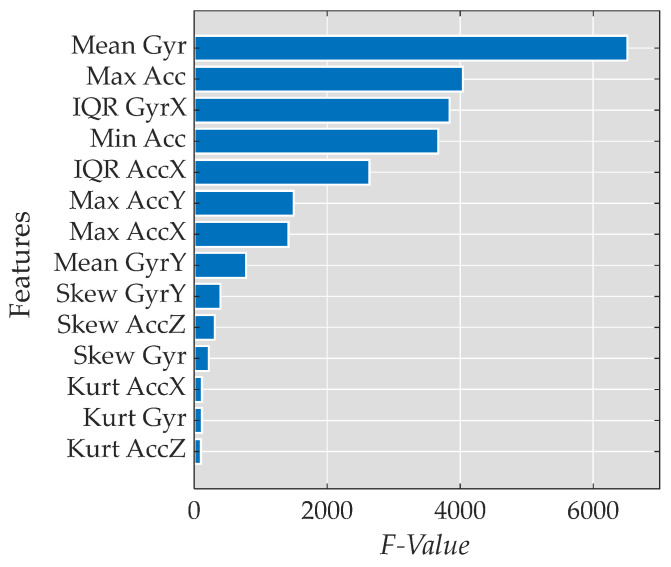
Bar plot presenting the *F*-Value of the selected features in descending order.

The analysis revealed that Heel Strike exhibited the highest values among the top four features when compared to the other gait phases or events. Additionally, all the features demonstrated different values for each phase within the 95% confidence interval (CI), except for Min |*Acc*|, which had the lowest *F*-Value among the top four features and showed the same values for both the Toe-Off event and Swing phase. Figure 4 presents the features in order of their relevance, based on the *F*-Value of the ANOVA test.

The identified features are shown in the following table (Table 2).

Among the identified features, the most important were as follows, described in Table 2 and named below:

1. Mean of the magnitude of the gyroscope data (Mean |Gyr|)
2. Maximum value of the magnitude of the acceleration data (Max |Acc|)
3. Interquartile range of the gyroscope data in the *x*-axis (IQR GyrX)
4. Minimum value of the magnitude of the acceleration data (Min |Acc|)

### 3.2. Spider Plots

**Figure 5:**
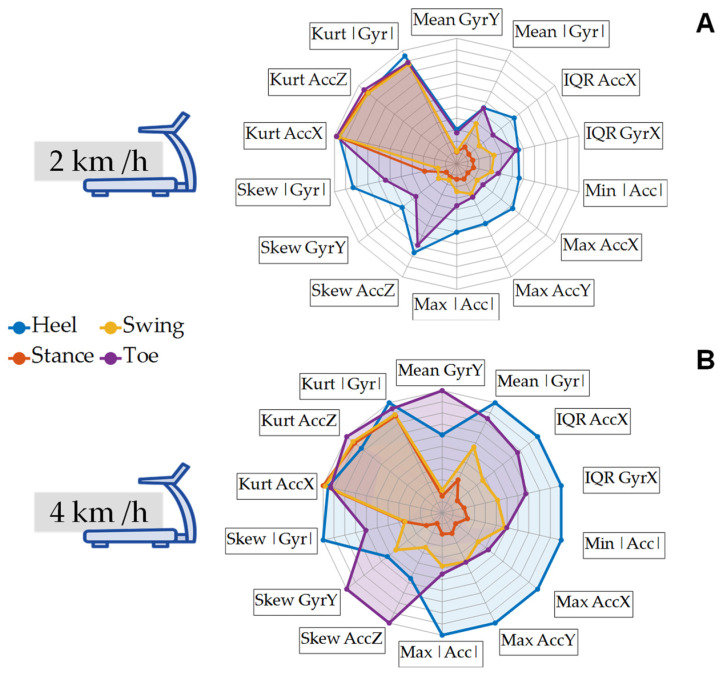
Spider plots illustrating the mean values of the selected features in the four studied categories: gait events (Toe-Off and Heel Strike) and phases (Stance and Swing) at different speeds: (**A**). 2 km/h and (**B**). 4 km/h.

Figure 5 presents a spider plot that showcases the selected features for gait analysis. The phases and events are represented by distinct colors: Heel Strike in blue, Stance in red, Toe-Off in purple, and Swing in yellow. Observations from the plot indicated that at 4 km/h, there was a clearer distinction between the gait events, compared to 2 km/h. The spider plots illustrates the gait pattern during the different events and phases at varying velocities. There was evidence of the clarity of distinguishing the gait events and phases at 4 km/h, compared to 2 km/h.

**Figure 6:**
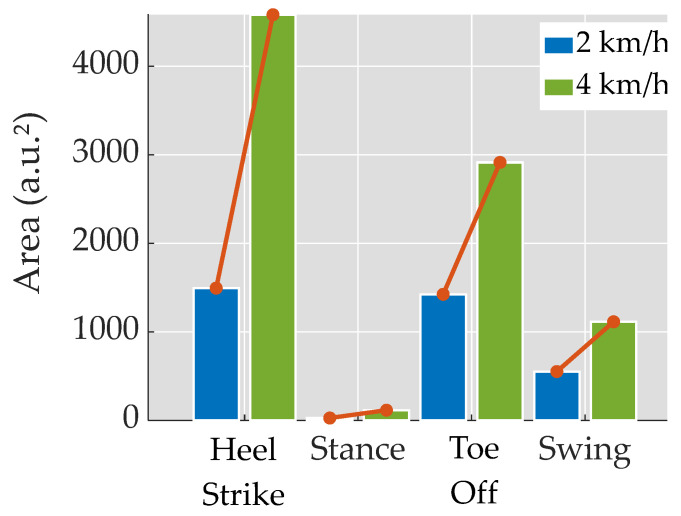
Bar plot displaying the area of the spider plot in the four studied categories: gait events (Toe-Off and Heel Strike) and phases (Stance and Swing) at 2 km/h (blue) and 4 km/h (green). The red lines connecting them serve as a graphical representation of the distinct ratios between the velocities, obtained by dividing the values from 4 km/h by those from 2 km/h.

Figure 6 illustrates the computed areas obtained from the spider plots for each phase and velocity. Notably, the areas exhibit an increase as the velocity rises. The ratios between the areas are as follows: Heel Strike = 3.07, Stance = 4.24, Toe-Off = 2.05, and Swing = 2.02. When ordered in descending order based on the area, the sequence is as follows: Heel Strike, Toe-Off, Swing, and Stance, at both velocities. Specifically, at 2 km/h, the areas for Toe-Off and Swing are approximately 1500 u.a.² each, indicating a similar magnitude for these two events and phases, respectively, at this velocity.

### 3.3. Machine Learning Analysis

**Figure 7:**
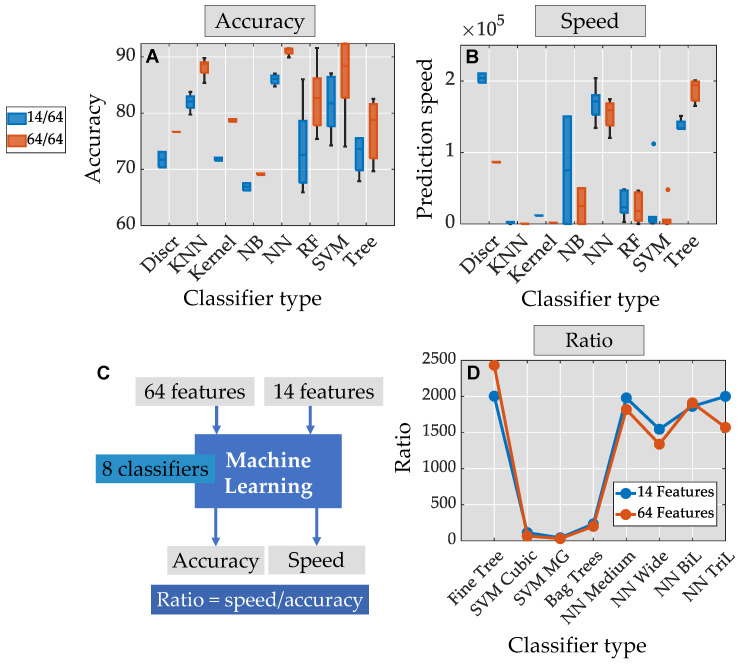
Boxplots illustrating the (**A**) performance and (**B**) prediction speed of all Machine Learning Models using the selected features. (**C**) Plot representing the ratio of Prediction Speed divided by Accuracy for Machine Learning Models with an accuracy higher than 85%, including Fine Tree due to its high Prediction Speed, using selected 14 features (blue) and 64 features (red). (**D**) Prediction speed/accuracy ratio, which indicates the trade-off between computational efficiency and model performance.

Figure 7A presents the accuracy results, while Figure 7B displays the prediction speed obtained from various types of algorithm models. A wider box plot indicates a greater dispersion in accuracy or prediction speed among algorithms from the same model type.

Regarding accuracy, Discriminant (Discr), Kernel, Naive Bayes (NB), and Neural Networks exhibit thin box plots, while Random Forest (RF), Support Vector Machine (SVM), and Decision Tree (Tree) show wide box plots. Additionally, using all features showed a greater accuracy compared to using only 14 features. In terms of prediction speed, Discr, KNN (K-Nearest Neighbors), Kernel, and SVM exhibited thin box plots, while NB, NN (Neural Networks), and RF had wider ones. Interestingly, the increase in the number of features results in decreased prediction speed in all models, except for Decision Trees. Specific attention has been directed towards certain algorithms (refer to Figure 7B and Table 3). The Fine Tree Algorithm displayed greater accuracy and prediction speed when additional features were integrated. On the other hand, SVM (Cubic and Gaussian) and Random Forest (Bagged Trees) exhibited a low ratio of Prediction Speed to Accuracy. In contrast, Neural Networks (NNs) exhibit a high ratio and achieve high absolute values in both Prediction and Accuracy.

Among the Neural Networks (NNs) tested, the NN Bilayered stood out with the highest ratio of accuracy to features reaching an impressive 91.5%. This indicates that the NN Bilayered achieved a high level of prediction accuracy while utilizing a relatively smaller number of features.

The Machine Learning analysis demonstrated that as the number of features increased, there was a corresponding increase in prediction speed. This trend is similar to what was observed with the Fine Tree Algorithm, indicating that increasing the number of features improved the prediction speed for both methods.

The implementation of a cubic kernel in the Support Vector Machine (SVM) resulted in the highest accuracy, reaching 92.3% when utilizing 64 features. However, the SVM Cubic still maintained a high accuracy of 87.1% even with a reduced feature set of 14 features.

## 4. Discussion

Recently, the study of human gait has become a topic of major interest within the field of human motor control. The analysis of gait could be a predictor of specific pathologies or could identify walking and posture problems, load anomalies, and muscle failure, which would not be measurable with normal clinical exams [8,23]. Another possible reason is the high human and economic cost of falling [13,14]. All this has led to numerous studies that describe the characteristics of normal gait using a multitude of methods [34,42,43], some of them using artificial intelligence [44], with the aim of being able to develop both injury prevention programs and orthoses to improve gait mechanics.

Spider plots, or radar chart plots, are valuable visualization tools that provide gait mapping in healthy young adults and offer a comprehensive overview of an individual’s gait pattern [45,46,47]. The use of spider plots in healthy adults seems to provide essential baseline data for gait analysis, helping to establish normal gait patterns and variations within this population [48]. They offer a promising approach for posterior pattern recognition in individuals with pathological conditions, allowing us to use them in clinical applications [46,49,50].

**Figure 8:**
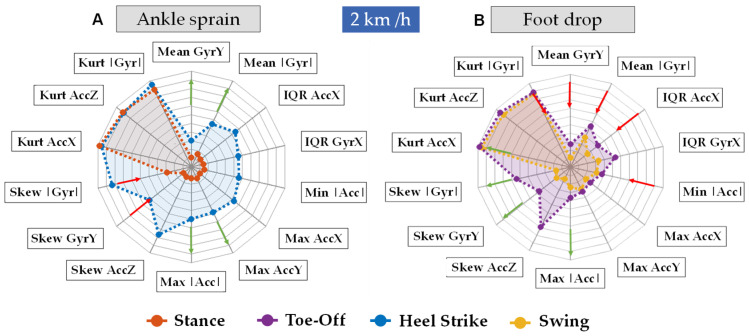
Spider plots illustrating the increase or decrease values of the selected features in the primary phases and events at 2 km/h: (**A**). Stance phase and Toe-Off event during foot drop and (**B**). Swing phase and Heel Strike event. The red and green arrows indicate a decrease or increase in the magnitude of the features respectively.

The best way to understand the benefits of the spider plot [51] for understanding gait pathology is by comparing normal versus pathologic (sprain and foot drop) plots (Figure 8). The same leg (right) for the normal and pathological data was plotted. In the absence of any impairments, we anticipate a consistent pattern of parameters across each specific phase or event. Nevertheless, pathological conditions may have the potential to alter the values of certain features during particular events or phases, which we will discuss further in this section.

Ankle sprains typically hold greater significance during the Stance Phase and the Heel Strike event, which occurs when the foot initially contacts the floor [3]. Conversely, conditions such as foot drop become more noticeable during the Swing Phase and the Toe-Off Event. This is often due to weakness in the anterior tibialis muscles or a lack of specificity in the plantar flexors during the Toe-Off Event [52].

The statistical feature assessing the maxima (Max) on the radar plot indicates the greatest achievable value of acceleration or rotation (gyroscope) during a specific time window within the treadmill test. The data from a patient with an ankle sprain showed Max AccY and Max GyrZ values, and consequently Max |Acc| and Max |Gyr|, their magnitude, would tend to increase significantly (see Figure 1C for axis reference), due to the inversion of the ankle.

The following paragraphs delve into the anticipated behavior of statistical features measured within the context of two pathological conditions: foot drop and ankle sprain. It is crucial to note that no specific tests have been conducted using the treadmill. Instead, the discussion is founded on the expertise of physiologists and the data collected from healthy subjects.

The statistical feature measuring the interquartile range (IQR) reveals the variability in acceleration or gyroscope measurements during testing. Foot drop tends to reduce IQR AccX and IQR Gyr Y, impacting overall magnitude, thereby leading to decreased IQR |Acc| and IQR |Gyr| due to limited dorsiflexion and resulting in reduced movement and a narrower motion range. Regarding ankle sprains, elevated maximum values can potentially indicate an increased IQR. However, the effect on the IQR depends on the sprain’s severity. Sharp and severe sprains, where multiple values spike, are likely to impact the IQR. In cases of rapid sprains with a brief peak and a quick return to normal gait, the IQR may remain relatively unaffected, particularly as values below the 75th percentile remain relatively unaffected.

The statistical feature known as kurtosis (Kurt) provides insights into the shape of the data. More precisely, low kurtosis suggests that there are numerous data points in the tails of the distribution, whereas high kurtosis indicates a concentration of data points in the central portion of the distribution. Variations in kurtosis values from those typically seen in normal gait analysis can indicate abnormalities in gait. Foot drop, which presents as inconsistent movement, can temporarily raise gyroscope values, leading to occasional outliers that diminish the Kurt |Gyr| feature. Furthermore, a decrease in acceleration along the *x*-axis can centralize the values, leading to an increase in Kurt AccX and, consequently, the Kurt |Acc| features.

The statistical feature skewness (Skew) quantifies the asymmetry of the data distribution. A positive acceleration skewness implies a right-skewed distribution, indicating a greater concentration of data points with lower acceleration values and fewer extremely high values. This trend could be indicative of a gait pattern characterized by slower movements, often observed in cases of foot drop. Conversely, a negative acceleration points to a left-skewed distribution, indicating more data points with higher acceleration values and fewer extreme low values. This might imply a gait pattern characterized by swifter or more abrupt movements, present during an ankle sprain.

Lastly, the statistical feature known as the mean (Mean) calculates the central values within a normal distribution. However, it can be influenced by outliers, making it less robust in such cases. In cases of foot drop, as per the hypothesis, a decrease in minimum values, a reduction in kurtosis, and maintaining the maximum in a positively skewed distribution are likely to result in an increased mean. Conversely, in the context of an ankle sprain, an increase in the maximum values and decrease of the skewness is expected to lead to an elevated mean, regardless of whether the interquartile range (IQR) increases or decreases.

It is worth highlighting that the present analysis of normal healthy individuals revealed four features to be the most relevant ones for the prediction of the two phases and two events of the gait (Heel Strike, Stance, Toe-Off, Swing). However, despite this finding, the integration of a more comprehensive set of features (14) has demonstrated its contribution to enhanced prediction accuracy [53]. This observation suggests that while certain features may play a prominent role in the predictive model, a broader set of features provides valuable additional information, leading to improved performance. Further studies should be conducted at higher velocities to gain a deeper understanding of the relationship between increased velocity and the algorithm’s performance in distinguishing the phases.

These results further reinforce the applicability of SVM in gait prediction [54,55,56], as well as Neural Networks [29,30]. Unlike other findings, NNs performed worse than SVM Cubic [57]. Greater accuracy indicates better performance of the algorithm in accurately predicting outcomes [58], while a greater prediction speed implies faster processing of predictions on test data. The results suggest that SVM Cubic can be a promising tool for diagnosis when prediction speed is not a determining factor. Meanwhile, NN Bilayered, which has high prediction speed and shows potential in recognizing events such as ankle sprains [59], is affected by time in gait recognition [60]. Liu et al. [61] observed worse ground reaction forces in patients after a lateral ankle sprain. These differences include greater reaction force in the midfoot and lateral forefoot [62], a laterally deviated center of pressure [63], and increased braking and propulsive forces [30] compared with uninjured participants. Fereydounnia et al. [64] found a worse reactivity and contraction of the peroneal musculature in patients who had suffered ankle sprains [32]. Therefore, it seems that the correct activation of this musculature is related to the prevention of ankle sprains by decreasing the inversion position [65,66]. With all this information, it seems essential to know the normal gait patterns and to have tools such as the one presented in this study for the detection of any abnormalities.

To draw definitive conclusions, further studies should explore the incorporation of more features, for instance, including spectral features, to refine and validate the results.

## 5. Limitations, Conclusions and Future Directions

The findings of this study should be interpreted within the context of its limitations. First, it is crucial to note that the study was conducted on a treadmill rather than in a real-world setting, such as on the streets. This distinction may influence the generalizability of the results to everyday situations. Moreover, there was a small sample. However, a significant number of variables have been analyzed to obtain the main characteristics of the gait. Additionally, the study included a limited number of subjects within a specific age range. This restricted age group might limit the broader applicability of the findings. To enhance the robustness of future research, we intend to focus on encompassing a more diverse range of age groups to understand potential variations in the observed outcomes better. A point to consider for future studies is the placement of the Arduino. Initially, it was secured on the forefoot of each participant using their respective shoelaces. However, starting from participant 20 onwards, we transitioned to using an elastic strap that encircles the foot for enhanced convenience and consistency.

In this study, an IMU unit was employed, considering the foot as a single segment under the rigid solid principle, which means that the foot is considered as singular entity when measuring its motion. Traditional kinematics mesurements of the multi-segment foot and ankle complex typically involve four segments (shank, hindfoot, forefoot, and toes) and are conducted using stationary motion capture devices. However, these devices are primarly confined to gait laboratories and are not practical for everyday clinical use. An alternative approach for assessing foot and ankle complex kinematics involves using an IMU attached to each segment using the algorithm proposed by Rouhani et al. [52].

In our case, for simplicity’s sake, we utilized a single IMU system. Although a single IMU unit may not provide a detailed diagnostic understanding of the underlying causes of foot abnormalities, using data from a single IMU on the foot segment alone offers a practical and less cumbersome method for assessing joint-level impairments in clinical practice. This is because the deviations in the foot segment detected by a single IMU system may be enough to discern the effects of conditions such as ankle sprains and foot drop pathologies.

This paper presents a preliminary study demonstrating a promising approach to gait recognition using spider plots as a diagnostic tool. The present results identify the optimal features of acceleration and gyroscope data during a normal gait. Using spider plots has provided valuable insights into gait mapping in healthy young adults, offering a comprehensive overview of their gait patterns. The creation of these spider plots is proposed to obtain a personalized fingerprint of each patient’s gait fingerprint, to be used as a diagnostic tool. A deviation from a normal gait pattern can be used to identify human gait disorders.

In summary, the statistical kinematics features in the spider plot can be helpful in the context of gait analysis for diseased individuals because:

- Identifying abnormal gait patterns: High kurtosis or significant skewness may serve as indicators of deviations from the typical gait patterns, potentially suggesting the presence of underlying pathologies or impairments in the kinematic movement of the subject.
- Quantifying variability: Both kurtosis and skewness can express how variable the acceleration data are.

Since excessive variability is linked to instability and a higher risk of falling in people with gait problems, it may be crucial to evaluate this aspect of gait data.

- Monitoring rehabilitation progress: These measures can also be used to track changes in gait over time. In the context of rehabilitation, a reduction in kurtosis and skewness may serve as an indicator of enhanced gait stability as the patient undergoes a rehabilitation intervention.

This information is valuable for advancing clinical applications in diagnosing gait-related disorders and developing novel orthoses to prevent falls and ankle sprains, since the alteration of the kinematic statistical parameters of the accelerometer and gyroscope will be proportional to the degree of severity of the subject’s pathology or injury.

## Figures and Tables

**Table 1:** Descriptive data of the participants (age, body mass, and body height) classified together by gender.

Variables	Gender Leg Dominance	N	Mean (SD)	CI 95%	Shapiro-Wilk W
Age (years)	Male	10	21.9 (1.85)	(20.6; 23.2)	0.873
	Female	22	22.2 (2.67)	(21.0; 23.4)	0.909
Body mass (kg)	Male	10	73.8 (5.87)	(69.6; 78.0)	0.953
	Female	22	59.9 (8.98)	(55.9; 63.9)	0.969
Body height (cm)	Male	10	183.2 (5.27)	(179.4; 187.0)	0.917
	Female	22	166.0 (4.31)	(164.0; 167.9)	0.968

**Table 2:** Descriptive statistics of the TOP 4 selected features divided by gait events.

Ranking	Features	Units *	Gait Event Phase	Mean (SD)	CI 95%
1	Mean |Gyr|	dps	Heel Strike	2 (34.6)	(75.0; 77.5)
			Stance	20.4 (20.7)	(20.2; 20.7)
			Toe-Off	47.6 (28.2)	(47.1; 48.1)
			Swing	67.2 (30.4)	(66.2; 68.4)
2	Max |Acc|	at	Heel Strike	0.44 (0.21)	(0.43; 0.45)
			Stance	0.08 (0.14)	(0.08; 0.09)
			Toe-Off	0.17 (0.12)	(0.17; 0.17)
			Swing	0.22 (0.12)	(0.21; 0.22)
3	IQR GyrX	dps	Heel Strike	61.0 (42.7)	(59.5; 62.5)
			Stance	10.8 (16.3)	(10.6; 11.0)
			Toe-Off	31.0 (28.8)	(30.5; 31.6)
			Swing	47.1 (33.2)	(45.9; 48.3)
4	Min |Acc|	at	Heel Strike	0.10 (0.07)	(0.10; 0.11)
			Stance	0.02 (0.03)	(0.02; 0.02)
			Toe-Off	0.05 (0.03)	(0.05; 0.05)
			Swing	0.05 (0.03)	(0.05; 0.05)

* dps: degress per second; at: atmospheres.

**Table 3:** Relevant parameters of the Machine Learning models with an accuracy higher than 85%, including Fine Tree due to its high Prediction Speed.

Model Type	Features	Accuracy	Prediction Speed [obs/s]
Fine Tree	16/64	75.5%	151,330
	64/64	82.5%	200,710
SVM Cubic	16/64	87.1%	9856
	64/64	92.4%	6100
SVM Median Gaussian	16/64	86.4%	3599
	64/64	92.3%	2772
Bagged Trees	16/64	86.0%	20,118
	64/64	91.6%	18,352
NN Medium	16/64	86.6%	171,405
	64/64	91.5%	166,463
NN Wide	16/64	87.1%	134,552
	64/64	89.9%	120,355
NN Bilayered	16/64	85.5%	159,197
	64/64	91.5%	174,773
NN Trilayered	16/64	86.1%	172,190
	64/64	91.3%	143,525

## Data Availability

Codes are available from the authors upon request.
